# Catheter-associated urinary tract infections in patients with gastric cancer after surgery: A retrospective study

**DOI:** 10.1097/MD.0000000000048533

**Published:** 2026-05-01

**Authors:** Jinlei Liu, Chunhai Zhang, Su Yan

**Affiliations:** a Graduate School of Qinghai University, Xining, Qinghai, China; b Gastrointestinal Oncology Surgery, Affiliated Hospital of Qinghai University, Xining, Qinghai, China.

**Keywords:** catheter-associated urinary tract infection, catheter duration, gastrectomy, gastric cancer, logistic regression, postoperative infection, retrospective study, urinary catheterization

## Abstract

Perioperative catheterization is a well-known risk factor for developing postoperative catheter-associated urinary tract infection (CAUTI). The information about the effect of extended urinary catheterization after surgery for gastric cancer is still scarce. This is a retrospective study of 232 patients with gastric cancer who underwent surgery. Patients were divided into 2 groups based on the duration of their catheter placement: <72 hours and 72 hours or more. The primary outcome was CAUTI during the index hospitalization. Logistic regression was used to calculate odds ratios in unadjusted, adjusted, and sensitivity models. A dose–response analysis was conducted for ordered catheter duration categories. In patients with catheterization duration ≥72 hours versus those with catheterization <72 hours, CAUTI occurred significantly more frequently. In the unadjusted model, prolonged catheterization remained associated with increased odds of CAUTI, odds ratio = 5.66. The relationship was still significant, adjusting for age, diabetes, and operative time (longer operation time). After omitting patients with shock or severe complications, long-term catheterization was still independently associated with CAUTI, with an odds ratio of 3.99. A significant duration-related trend was observed with a steep increase in CAUTI after 72 hours of catheterization. Urinary catheterization for ≥72 hours was associated with an increased risk of CAUTI after gastric cancer surgery, which warrants the avoidance of unnecessary catheter prolongation if clinically possible.

## 1. Introduction

Gastric cancer is still a major public problem and contributes high numbers to cancer-related death globally.^[1,2]^ Even with the development of systemic treatment, surgery remains the basis for potential cure in localized and resectable disease, and modern guidelines consider gastrectomy with appropriate lymphadenectomy as a crucial element of the standardized care pathway.^[3]^

Postoperative complications following gastrectomy are frequent and have a burden of clinical significance. In a population-based national cohort of patients receiving gastrectomy for gastric adenocarcinoma, complications were frequent both at 30 and 90 days after surgery, and infectious complications made up a large part of the postoperative complications.^[4]^ Furthermore, apart from completing the short-term recovery, accumulated studies have indicated that postoperative complications, especially infectious complications, hurt the long-term outcome after gastrectomy, including overall and cancer-specific survival.^[5–7]^ These results taken together emphasize a strong potential for perioperative modifiable factors to reduce infection risk and improve outcomes after surgery for gastric cancer.

With the goal of minimizing physiologic perturbation, promoting early return of function, and reducing length of hospital stay, enhanced recovery after surgery (ERAS) protocols have recently been more widely applied to gastrectomy. The ERAS Society consensus guidelines for gastrectomy introduce an evidence-based approach to perioperative care, device management included, that facilitates early mobilization and reduces exposure to the catheter.^[8,9]^ Some recent meta-analyses and randomized studies of gastric cancer surgery also demonstrate that ERAS-based management improves short-term recovery without an increase in overall morbidity if appropriately performed, even if the practice compliance with several items differs by center and patient cohort.^[10–12]^

Indwelling urinary catheterization is a standard perioperative routine in major abdominal surgery, but extended catheter exposure is a major contributor to catheter-associated urinary tract infection (CAUTI), one of the most frequent healthcare-associated infections.^[13]^ Interventions aimed at decreasing catheter dwell time, including protocols and nurse-driven strategies, have been linked with reductions in catheter utilization and CAUTI in multiple inpatient areas.^[14]^ In gastrointestinal surgery populations, randomized evidence and observational prediction studies also highlight the clinical tradeoff between earlier catheter removal and risks such as urinary retention and recatheterization while repeatedly identifying catheterization duration as an important determinant of postoperative urinary tract infection risk.^[15,16]^ However, gastric cancer-specific data that directly quantify CAUTI risk across clinically relevant catheter-duration thresholds remain limited in routine practice cohorts, and the real-world balance between prolonged catheterization and downstream infectious outcomes is incompletely characterized.

Therefore, this retrospective study evaluated CAUTIs in patients undergoing gastric cancer surgery, focusing on the association between prolonged postoperative catheterization and CAUTI risk. We further aimed to describe clinical characteristics linked to prolonged catheter use and to generate clinically interpretable estimates that can inform perioperative catheter management in routine gastric cancer surgical care.

## 2. Materials and methods

### 2.1. Study design and setting

This study was approved by the Ethics Committee of the Affiliated Hospital of Qinghai University. This retrospective observational study evaluated CAUTIs in patients who underwent surgery for gastric cancer. Clinical and laboratory data were extracted from routinely collected electronic medical records at the Affiliated Hospital of Qinghai University Xining. All included patients had stage III gastric cancer. This restriction was applied to improve cohort homogeneity, as stage III patients typically undergo more standardized radical gastrectomy and have relatively comparable perioperative risk profiles. By limiting disease stage variability, we aimed to reduce confounding related to differences in tumor burden, surgical extent, and treatment strategy.

### 2.2. Study population

#### 2.2.1. Inclusion criteria

Patients were included if they met all of the following criteria:

1. Adult patients who underwent gastrectomy for gastric cancer during the study period.
2. Postoperative indwelling urinary catheterization was performed, and both catheter insertion time and catheter removal time were documented to allow calculation of catheter duration.
3. Availability of essential perioperative and outcome data required for analysis, including CAUTI occurrence during the index hospitalization.
4. Availability of postoperative laboratory data on postoperative day (POD) 1 and 3, including white blood cell (WBC) count and C-reactive protein (CRP).
5. All included patients had stage III gastric cancer according to the staging records in our center.

#### 2.2.2. Exclusion criteria

Patients were excluded if they met any of the following criteria:

1. Missing or implausible catheter timing records that prevented calculation of catheter duration.
2. Evidence of urinary tract infection at admission or before catheterization, or ongoing antibiotic treatment for a documented urinary tract infection prior to surgery.
3. Concomitant urological surgery or preexisting long-term urinary catheter dependence prior to admission.
4. Missing primary outcome ascertainment for CAUTI during hospitalization.
5. Reoperation during the index admission before catheter removal, if catheter duration could not be attributed to the index surgery course.

### 2.3. Exposure definition and grouping strategy

The exposure of interest was postoperative indwelling urinary catheter duration. Catheter duration was calculated as the time between catheter insertion and catheter removal recorded in the medical chart. Patients were categorized into 2 groups for the main analysis. The one is short catheterization group, which was defined as catheter duration <72 hours, while the other prolonged catheterization group was defined as catheter duration ≥72 hours. For dose–response analyses, catheter duration was additionally categorized into 4 ordered levels: <48 hours, 48 hours to <72 hours, 72 hours to <96 hours, and ≥96 hours.

### 2.4. Outcome definitions

The primary outcome was CAUTI. CAUTI events were identified from the clinical record based on the treating team’s diagnosis of urinary tract infection occurring in the setting of an indwelling urinary catheter, with event timing during catheterization or shortly after catheter removal, consistent with widely used surveillance frameworks.

Secondary outcomes included urine culture testing and culture positivity, postoperative fever, maximum temperature, postoperative inflammatory markers, and length of stay. Urine culture positivity was defined as a positive microbiological culture result when a urine specimen was submitted. Because urine cultures were ordered at clinician discretion, culture testing frequency and positivity were reported separately from CAUTI classification.

The primary outcome was CAUTI. CAUTI events were identified from clinical records based on physician diagnosis of urinary tract infection occurring in the presence of an indwelling urinary catheter or within a short period after catheter removal. In our institution, diagnoses were made according to routine clinical practice, which is broadly consistent with established surveillance frameworks such as the Centers for Disease Control and Prevention/National Healthcare Safety Network criteria, incorporating clinical symptoms, laboratory findings, and, when available, microbiological evidence. Diagnostic practices were applied consistently across the study period.

### 2.5. Covariates and perioperative variables

In this study, the demographic features for each included patient were collected and analyzed: sex, age, body mass index, American Society of Anesthesiologists class, and whether or not to have diabetes mellitus, hypertension, and chronic kidney disease. Operative and perioperative variables were surgical approach, radical extent, operative time, estimated blood loss, intraoperative transfusion, postoperative fluid volume in the first 24 hours, intensive care unit (ICU) admission, and postoperative situations that possibly necessitate prolonged catheterization, including hemodynamic instability or shock and major complications. Catheter-related events included recatheterization and urinary retention, and the laboratory indices included WBC count as well as CRP measured on POD 1 and 3 according to routine clinical practice. Major complications were defined according to the Clavien–Dindo classification as grade III or higher complications requiring surgical, endoscopic, or radiological intervention, or resulting in life-threatening conditions.

### 2.6. Statistical analysis

Continuous variables are presented as mean and standard deviation or median and interquartile range, as appropriate. Categorical variables are presented as counts and percentages. Comparisons between groups were conducted using the Student *t* test or Mann–Whitney *U* test for continuous variables, and the Pearson chi-square or Fisher exact test for categorical variables. Duration of catheterization and CAUTI were related using binary logistic regression to calculate odds ratios with 95% confidence intervals, considering a crude model and an adjusted model.

Besides, the sensitivity analysis was conducted by repeating the adjusted model after excluding patients with shock or major complications. A trend analysis across ordered catheter duration categories was performed using logistic regression with the ordinal exposure entered as a continuous term. For the above analysis, a two-sided *P* < .05 was considered statistically significant, and the analyses were conducted using IBM SPSS Statistics version 29.0.

## 3. Results

### 3.1. Study population and baseline characteristics

A total of 232 postoperative gastric cancer patients were included and were categorized into a short catheterization group and a prolonged catheterization group according to catheter duration (Table 1). Several baseline characteristics were similar between groups; however, patients in the prolonged catheterization group were older, had a higher body mass index, and more frequently had hypertension.

**Table 1:** Baseline characteristics of patients according to catheterization group.

Variable	Short catheterization (<72 h, n = 127)	Prolonged catheterization (≥72 h, n = 105)	*P* value
Age, yr	57.16 ± 10.14	63.48 ± 10.84	<.001
BMI, kg/m^2^	24.17 ± 3.15	25.26 ± 3.05	.008
Sex			
Female	63 (49.6%)	51 (48.6%)	.875
Male	64 (50.4%)	54 (51.4%)	
ASA class			
ASA I	17 (13.4%)	14 (13.3%)	.378
ASA II	52 (40.9%)	32 (30.5%)	
ASA III	49 (38.6%)	51 (48.6%)	
ASA IV	9 (7.1%)	8 (7.6%)	
Diabetes mellitus	32 (25.2%)	27 (25.7%)	.928
Hypertension	56 (44.1%)	61 (58.1%)	.034
Chronic kidney disease	9 (7.1%)	14 (13.3%)	.113

ASA = American Society of Anesthesiologists, BMI = body mass index.

### 3.2. Operative and early postoperative characteristics

As shown in Table 2, operative and perioperative characteristics differed between groups. The prolonged catheterization group underwent more extensive radical procedures and showed greater operative burden, reflected by longer operative time and higher estimated blood loss. Postoperative resource use was also higher in the prolonged catheterization group, including more frequent transfusions and ICU admission. Major complications were more common in the prolonged catheterization group, while hemodynamic instability or shock did not differ significantly between groups.

**Table 2:** Operative and early postoperative characteristics by catheterization group.

Variable	Short catheterization (<72 h, n = 127)	Prolonged catheterization (≥72 h, n = 105)	*P* value
Surgical approach			
Open	31 (24.4%)	40 (38.1%)	.098
Laparoscopic	67 (52.8%)	41 (39.0%)	
Robotic	26 (20.5%)	20 (19.0%)	
Other	3 (2.4%)	4 (3.8%)	
Radical extent			
Standard radical	106 (83.5%)	53 (50.5%)	<.001
Extended radical	18 (14.2%)	41 (39.0%)	
Other	3 (2.4%)	11 (10.5%)	
Operative time, min	148.61 ± 36.63	218.99 ± 59.17	<.001
Estimated blood loss, mL	313.96 ± 144.23	529.05 ± 225.25	<0.001
Postoperative fluid volume (first 24 h), mL	2454.41 ± 485.92	3147.08 ± 680.21	<.001
Blood transfusion	16 (12.6%)	33 (31.4%)	<.001
ICU admission	10 (7.9%)	29 (27.6%)	<.001
Hemodynamic instability/shock	4 (3.1%)	5 (4.8%)	.735
Major complications	15 (11.8%)	27 (25.7%)	.010

ICU = intensive care unit.

### 3.3. Catheterization profile and catheter-related events

Catheter duration was longer in the prolonged catheterization group by definition. Catheter-related adverse events were more frequent in the prolonged catheterization group, including higher rates of recatheterization and urinary retention. The catheterization profile and catheter-related events are presented in Table 3.

**Table 3:** Catheterization profile and catheter-related events.

Variable	Short catheterization (<72 h, n = 127)	Prolonged catheterization (≥72 h, n = 105)	*P* value
Catheter duration, h	48.94 ± 13.09	103.09 ± 25.01	<.001
Catheter duration category			
<48 h	61 (48.0%)	0 (0.0%)	<.001
48–<72 h	66 (52.0%)	0 (0.0%)	
72–<96 h	0 (0.0%)	42 (40.0%)	
≥96 h	0 (0.0%)	63 (60.0%)	
Recatheterization	10 (7.9%)	27 (25.7%)	<.001
Urinary retention	10 (7.9%)	17 (16.2%)	.039

### 3.4. Clinical outcomes and microbiological findings

The prolonged catheterization group experienced a higher incidence of CAUTI. Urine culture sampling was performed more often in the prolonged catheterization group, and overall culture positivity was higher at the cohort level. Among patients who underwent urine culture testing, the culture positivity rate did not differ significantly between groups (Table 4). Postoperative inflammatory response was more pronounced in the prolonged catheterization group, with higher inflammatory markers on POD 1 and POD 3, higher maximum temperature, and more frequent fever. Length of hospital stay was also longer in the prolonged catheterization group.

**Table 4:** Clinical outcomes and microbiological findings according to catheterization group.

Variable	Short catheterization (<72 h, n = 127)	Prolonged catheterization (≥72 h, n = 105)	*P* value
CAUTI	6 (4.7%)	23 (21.9%)	<.001
Urine culture obtained	35 (27.6%)	57 (54.3%)	<.001
Urine culture positive (overall)	10 (7.9%)	26 (24.8%)	<.001
Culture positivity among those tested	10 (28.6%)	26 (45.6%)	.104
Colony count among culture-positive, median (IQR)	1.68 × 106 (1.28 × 106, 4.29 × 106)	4.96 × 106 (2.60 × 106, 1.55 × 107)	
WBC on POD1, 10^9^/L	10.99 ± 2.19	12.32 ± 2.92	<.001
CRP on POD1, mg/L	84.80 ± 28.43	100.00 ± 36.21	<.001
WBC on POD3, 10^9^/L	9.82 ± 2.28	11.32 ± 3.16	<.001
CRP on POD3, mg/L	68.10 ± 24.83	100.13 ± 41.21	<.001
Maximum temperature, °C	37.41 ± 0.59	37.74 ± 0.73	<.001
Length of stay, d	8.11 ± 2.45	13.66 ± 4.49	<.001
Fever	20 (15.7%)	29 (27.6%)	.035

CAUTI = catheter-associated urinary tract infection, CRP = C-reactive protein, IQR = interquartile range, POD = postoperative day, WBC = white blood cell.

### 3.5. Dose–response relationship and regression analyses

A significant dose–response relationship between catheter duration and CAUTI was observed (Table 5), with a marked increase in incidence once catheterization exceeded 72 hours. In regression analyses (Table 6), prolonged catheterization remained significantly associated with increased odds of CAUTI in both unadjusted and adjusted models. The association persisted in sensitivity analyses excluding patients with shock or major complications.

**Table 5:** Dose–response relationship between catheter duration categories and CAUTI incidence.

	N	CAUTI, n (%)	OR vs <48 h (95% CI)	*P* value
Catheter duration category				
<48 h	61	4 (6.6%)	Reference	NA
48–72 h	66	2 (3.0%)	0.45 (0.08–2.52)	.361
72–96 h	42	9 (21.4%)	3.89 (1.11–13.61)	.034
≥96 h	63	14 (22.2%)	4.07 (1.26–13.18)	.019
Overall association (Pearson χ^2^, df = 3)				.005
*P* for trend (ordinal categories)				.001

CAUTI = catheter-associated urinary tract infection, CI = confidence interval, OR = odds ratio.

**Table 6:** Sensitivity analyses and parsimonious logistic regression models for CAUTI.

Analysis/model	Covariates	Effect estimate	*P* value	N (events)
Unadjusted	Group only	5.66 (2.21–14.50)	<.001	232 (29)
Adjusted model 1	Group + age + diabetes + operative time	5.68 (1.87–17.26)	.002	232 (29)
Sensitivity (exclude shock/major)	Group + age + diabetes + operative time	3.99 (1.17–13.54)	.027	183 (22)
Ordinal exposure model	Catheter category (per 1-level increase) + age + diabetes + operative time	1.85 (1.16–2.93)	.009	232 (29)

CAUTI = catheter-associated urinary tract infection.

## 4. Discussion

In this retrospective cohort of patients undergoing surgery for gastric cancer, prolonged postoperative catheterization of at least 72 hours was associated with a higher risk of CAUTI. In the group comparison, the prolonged catheterization group showed a substantially higher CAUTI incidence than the short catheterization group. The association remained statistically significant in parsimonious regression models and persisted after excluding patients in whom shock or major complications could mandate extended catheterization, although the effect size was attenuated. These findings collectively support the interpretation that catheter duration is a clinically relevant and potentially modifiable factor associated with postoperative CAUTI in this surgical population.

The relationship observed is biologically plausible and is consistent with the known epidemiology of CAUTI, where catheter days are universally ranked as a leading risk factor.^[17,18]^ From a mechanistic standpoint, indwelling catheters serve as a substrate on which bacteria can adhere and form biofilms that are less susceptible to host defenses and antimicrobials and that facilitate ascending infection with continued device exposure.^[19–21]^ Traditional and recent reviews point out that a great percentage of catheter-associated bacteriuria or CAUTI events are paucisymptomatic, and clinician detection may be limited without systematic evaluation, reiterating the importance of uniform definitions and surveillance in retrospective studies.^[22,23]^

Beyond the dichotomous exposure definition, the dose–response analysis in this study provides additional support for a duration-dependent association. When catheterization was categorized, CAUTI incidence remained low in categories below 72 hours and increased sharply in categories ≥72 hours, with a statistically significant trend across ordered categories. Moreover, an ordinal exposure model demonstrated that each step increase in the catheter duration category was associated with higher odds of CAUTI after adjustment. Duration-dependent gradients are frequently highlighted in the broader CAUTI literature, including meta-analytic evidence that prolonged catheterization is a consistent risk factor across settings.^[18,24,25]^

A clinically important consideration is confounding by indication, because patients with more complex perioperative courses often require prolonged catheterization. In this cohort, the prolonged catheterization group had signals of higher surgical burden and postoperative intensity of care, including longer operative time, greater blood loss and transfusion, more ICU admission, and higher major complication rates. Together, these features are likely to increase both the demand for prolonged catheterization and the baseline vulnerability to infection through systemic stress response, immobilization, and rival infectious or inflammatory syndromes. Nonetheless, the persistence of the association in adjusted and restricted analyses indicates that catheter duration may provide unique information about CAUTI risk rather than being a proxy for severity.

Catheter-related events observed in the prolonged group also inform practical prevention priorities. Recatheterization and urinary retention were more common among patients with prolonged catheterization. This clustering matters because reinsertion can disrupt closed drainage systems and may introduce additional opportunities for microbial contamination, while urinary retention can prompt prolonged device dependence and repeated instrumentation. Studies in ERAS contexts, particularly colorectal surgery pathways with early Foley removal, illustrate the clinical trade-off whereby earlier removal may increase postoperative urinary retention and intermittent catheterization while not necessarily increasing CAUTI during the index admission.^[26]^ Therefore, the optimal strategy is rarely a single timing rule. Instead, a structured pathway that couples early removal criteria with standardized bladder assessment and reinsertion thresholds is more likely to reduce both prolonged exposure and unnecessary recatheterization.

The findings are also relevant to ERAS implementation in gastrectomy, where compliance with urinary catheter management can be variable and may be lower in clinically vulnerable subgroups.^[27,28]^ A recent randomized trial in radical gastrectomy evaluated immediate catheter removal at the end of surgery versus removal within 24 hours under ERAS care and reported non-inferiority for urinary retention, suggesting that earlier removal can be feasible when supportive perioperative elements are optimized.^[29]^ Although the exposure threshold in our study focuses on durations ≥72 hours rather than the first POD, the trial evidence supports a general principle that earlier removal can be safe in selected gastrectomy patients, potentially preventing unnecessary extension into higher-risk catheter-day ranges.

Microbiological data in this cohort should be interpreted in the context of differential testing intensity. Urine cultures were obtained more frequently in the prolonged catheterization group, and overall culture positivity was higher. However, among those who were tested, the positivity proportion did not differ significantly between groups. This pattern is compatible with clinician-driven sampling, where perceived risk or symptoms may prompt testing more often in patients with prolonged catheterization. Future prospective work could reduce this bias by applying standardized triggers for urine culture and anchoring CAUTI case definitions to widely accepted guideline criteria.^[30]^

Inflammation-related indices and clinical recovery markers provide further context for the postoperative course. The prolonged catheterization group showed elevated WBC and CRP on POD 1 and 3, higher maximum temperature, a higher fever rate, and longer hospital stay. These contrasts may reflect several intertwined mechanisms, including greater operative stress, a higher rate of complications, and the implication of infections such as CAUTI in systemic inflammation. As retrospective data cannot be expected to accurately establish temporality between catheter duration, systemic inflammation, and infection onset in every subject, these observations should be viewed as associated characteristics of a more complex postoperative course rather than as signs of direct causation.

Despite the strengths of this study, several limitations should be acknowledged. First, the retrospective observational design precludes causal inference. Although we adjusted for key clinical variables such as age, diabetes, and operative time, residual confounding from unmeasured factors cannot be excluded. In particular, variables such as frailty status, nutritional condition, baseline immune function, and perioperative functional reserve were not systematically captured in our dataset. These factors may influence both the likelihood of prolonged catheterization and susceptibility to infection, potentially biasing the observed association. Therefore, our findings should be interpreted as demonstrating an association rather than a causal relationship. In addition, although CAUTI ascertainment was broadly aligned with Centers for Disease Control and Prevention/National Healthcare Safety Network frameworks, the lack of strict prospective adjudication using standardized criteria may introduce outcome misclassification. The restriction to stage III gastric cancer patients may limit the generalizability of our findings. Patients with earlier-stage disease or metastatic disease may have different perioperative courses, surgical complexity, and baseline risk of infection. Therefore, extrapolation of these findings to all gastric cancer populations should be made with caution, and future studies including broader disease stages are warranted.

## 5. Conclusion

In conclusion, longer duration of catheterization ≥72 hours was associated with a greatly increased risk of developing a CAUTI after gastrectomy, with a clear duration-dependent trend indicated by a sharp rise in CAUTI when catheterization exceeded 72 hours. These results encourage perioperative interventions for reducing catheter exposure when clinically appropriate, and for structured management of urinary retention to avert unnecessary recatheterization and consequent advancement into greater-risk catheter day intervals.

## Author contributions

**Conceptualization:** Jinlei Liu, Chunhai Zhang, Su Yan.

**Data curation:** Jinlei Liu, Chunhai Zhang, Su Yan.

**Formal analysis:** Jinlei Liu, Chunhai Zhang, Su Yan.

**Funding acquisition:** Jinlei Liu, Su Yan.

**Investigation:** Su Yan.

**Writing – original draft:** Jinlei Liu, Su Yan.

**Writing – review & editing:** Jinlei Liu, Su Yan.
